# DeepRisk network: an AI-based tool for digital pathology signature and treatment responsiveness of gastric cancer using whole-slide images

**DOI:** 10.1186/s12967-023-04838-5

**Published:** 2024-02-19

**Authors:** Mengxin Tian, Zhao Yao, Yufu Zhou, Qiangjun Gan, Leihao Wang, Hongwei Lu, Siyuan Wang, Peng Zhou, Zhiqiang Dai, Sijia Zhang, Yihong Sun, Zhaoqing Tang, Jinhua Yu, Xuefei Wang

**Affiliations:** 1 Department of Gastrointestinal Surgery, Zhongshan Hospital, Fudan University, 180 Fenglin Road, Shanghai, 200032 China; 2 Gastric Cancer Center, Zhongshan Hospital, Fudan University, Shanghai, China; 3 Cancer Center, Zhongshan Hospital, Fudan University, Shanghai, China; 4 Biomedical Engineering Center, School of Information Science and Technology, Fudan University, Shanghai, 200433 China; 5 The Key Laboratory of Medical Imaging Computing and Computer Assisted Intervention of Shanghai, Shanghai, China; 6 Department of Immunology and Pathogenic Biology, School of Basic Medical Sciences, Shanghai University of Traditional Chinese Medicine, Shanghai, People’s Republic of China; 7 Department of General Surgery, Zhongshan Hospital (Xiamen), Fudan University, Xiamen, China; 8 Xiamen Clinical Research Center for Cancer Therapy, Zhongshan Hospital (Xiamen), Fudan University, Xiamen, China

**Keywords:** Deep learning, Gastric cancer, Whole slide image, Artificial intelligence, Suppressive immune microenvironment

## Abstract

**Background:**

Digital histopathology provides valuable information for clinical decision-making. We hypothesized that a deep risk network (DeepRisk) based on digital pathology signature (DPS) derived from whole-slide images could improve the prognostic value of the tumor, node, and metastasis (TNM) staging system and offer chemotherapeutic benefits for gastric cancer (GC).

**Methods:**

DeepRisk is a multi-scale, attention-based learning model developed on 1120 GCs in the Zhongshan dataset and validated with two external datasets. Then, we assessed its association with prognosis and treatment response. The multi-omics analysis and multiplex Immunohistochemistry were conducted to evaluate the potential pathogenesis and spatial immune contexture underlying DPS.

**Results:**

Multivariate analysis indicated that the DPS was an independent prognosticator with a better C-index (0.84 for overall survival and 0.71 for disease-free survival). Patients with low-DPS after neoadjuvant chemotherapy responded favorably to treatment. Spatial analysis indicated that exhausted immune clusters and increased infiltration of CD11b^+^CD11c^+^ immune cells were present at the invasive margin of high-DPS group. Multi-omics data from the Cancer Genome Atlas-Stomach adenocarcinoma (TCGA-STAD) hint at the relevance of DPS to myeloid derived suppressor cells infiltration and immune suppression.

**Conclusion:**

DeepRisk network is a reliable tool that enhances prognostic value of TNM staging and aid in precise treatment, providing insights into the underlying pathogenic mechanisms.

**Supplementary Information:**

The online version contains supplementary material available at 10.1186/s12967-023-04838-5.

## Introduction

Gastric cancer (GC) is one of the most lethal malignancies worldwide with poor prognosis [1]. It is extensively heterogeneous at the histological, molecular and genetic levels, making it challenging to treat effectively [2]. Radical gastrectomy remains the mainstay of curative treatment for GCs. Unfortunately, the prognosis after surgery is unsatisfactory due to a high incidence of recurrence and/or distant metastases [3]. Therefore, improvements in the identification of patients with poor clinical outcomes are needed for the optimization of therapeutic strategies and better allocation of adjuvant therapies.

In past decades, growing evidences proved that tumour microenvironment and tumour biology behavior play an important role in gastric cancer progression and prognostic prediction, such as the four-factor immunoscore system [4] and seven-gene signature [5]. However, in clinical practice, these clinical tests are costly and time-consuming, limiting their clinical applications in all patients. Recently, new pathological features such as collagen signature [6], tertiary lymphoid structures [7] and high endothelial venules [8] have been investigated as novel prognostic factors, indicating that pathomics of cancer tissues contain a wealth of information that can provide benefits to the patient beyond diagnosis [9]. Further understanding of the prognostic value of GC pathological features may facilitate prognostic stratification and the establishment of treatment strategies in clinical practice.

Deep learning has made it possible to perform complex analysis of high-resolution histopathology images and to explore the potential correlations between pathological factors and carcinogenesis in various types of cancer, such as colorectal cancer [10] and liver cancer [11, 12]. Accumulating evidence indicates that deep learning algorithms applied to routine histological data can help diagnose origins of cancers [13], detect Epstein–Barr virus status in gastric cancer [14], and predict the therapeutic response [15]. Recently, Wang et al. showed that deep learning could also be used for histological grading based on whole-slide images (WSIs) in breast cancer [16]. Studies have also proven that deep learning is able to identify significant prognostic factors from digital WSIs [11], and to assist in the allocation of adjuvant therapies to patients with a high risk of recurrence in primary melanoma tumours [17]. With respect to GC, a deep learning-based approach based on ResNet18 was applied to hematoxylin and eosin (H&E)-stained histopathological slides of tissue samples from GC patients and allowed the prediction of microsatellite instability (MSI) [18]. A more recent study applied nomogram to build a machine learning model to predict GC prognosis, which used randomly selected image patches from WSI for modelling [19]. However, the existing methods for WSI analysis faces many limitations. For methods that identify different types of tissue regions, manual annotation is required and therefore time-consuming and subjective, leading to reduced generalization performance [12, 13, 20]. Methods that require manual design of features are often suboptimal because the designed features lack generalization and are not very expressive [17, 19]. In addition, most models lack the fusion of features at different magnification, which is critical for the pathologist's examination [14–16]. Further, most artificial intelligence (AI)-based pathology biomarkers mainly focused on different aspects of prediction, lacking the exploration of underlying biological mechanism.

In the present study, we sought to establish a fully automated histopathological slide analysis pipeline that can use original scanned WSIs as input, automatically locate the tissue area, and output a risk score without any manual annotation. We established a deep risk network (DeepRisk) to obtain a novel digital pathology signature (DPS) as the model output using WSIs from the Zhongshan dataset, and then validated the model performance using 2 external datasets. To explore the underlying pathogenic mechanism and clinical significance of DPS, we also investigated the relationships of the DPS with tumour immune infiltration, spatial immune contexture, as well as treatment response of neoadjuvant treatment.

## Materials and methods

### Datasets

In our study, we developed a dataset for training and testing the DeepRisk model and determining the DPS values. A total of 1120 patients with a pathologically confirmed diagnosis of GC who underwent surgical resection between April 2006 and November 2019 at Zhongshan Hospital of Fudan University (Shanghai, China) were enrolled in this study. Eligible patients were required to have clinicopathological data and follow-up data, not to have any other malignancies.

The Cancer Genome Atlas (TCGA) and Shanghai Outdo Biotech Company (SOBC) datasets were used as external independent datasets for validation of the deep learning model. We collected clinical, pathological and prognostic factor data for 268 patients with OS ≥ 1 month in the TCGA–stomach adenocarcinoma (STAD) dataset and downloaded the associated WSIs from the public website (https://portal.gdc.cancer.gov/projects/TCGA-STAD) [20]. After excluding WSIs with poor image quality (such as poor staining or tissue overlap), 536 WSIs were available for further analysis. The SOBC dataset, obtained from SOBC with the approval of the Institutional Review Board, only consisted of tissue microarray (TMA) spots. Paired TMAs of tumour and normal tissues from 277 GC patients were scanned using a Hamamatsu (Hamamatsu Photonics, Hamamatsu City, Japan) scanner at 20 × magnification. All available core samples from each patient were used for analysis as previously described [21]. Due to a lack of tumour recurrence information in the TCGA-STAD and SOBC datasets, OS was used as the primary outcome measure in the survival analysis during model validation.

### Tumour characterization and follow-up

Tumour sample collection, postoperative surveillance, adjuvant treatment, and recurrence management were performed as previously described [22]. Tumours were staged according to the 8th edition of the Union for International Cancer Control (UICC)/TNM classification [23]. GC patients were stratified according to tumour differentiation (differentiated or undifferentiated), tumour stage (I-II or III-IV), and adjuvant treatment (received or not received) [24]. The classification of pathological tissue regression after neoadjuvant chemotherapy depends on Ninomiya and Ryan classification systems [25, 26]. The follow-up duration was measured from the time of surgery to the last follow-up date, and patient survival information was collected at the last follow-up. Patients who had not experienced recurrence or were alive at the last follow-up were censored at the last follow-up.

### Slide preparation

H&E-stained histopathological slides were prepared as previously described [27]. All slides in the Zhongshan dataset were scanned and digitized using Aperio (Leica Biosystems, Wetzlar, Germany) and Hamamatsu (Hamamatsu Photonics, Hamamatsu­City, Japan) digital slide scanners at 40× magnification. One or two representative digital slides were selected and analysed for each GC patient.

### WSI processing

A threshold-based algorithm was applied for the detection and segmentation of stained tumour tissue on digitized slides [28], which were processed after 8 × downscaling due to the considerable heterogeneity in resolution and computational processing. Tissue regions in the approximate contour obtained through segmentation were exhaustively tiled with 256 × 256 patches at different magnification levels (5× , 10× and 20×). The overlap ratio between adjacent patches was set to 0 at each magnification level during model training, testing and validation, but an overlap ratio of 50% was applied to improve the resolution for feature visualization.

To reduce the computational complexity and extract the information in the image patches, a deep convolutional neural network (CNN) was firstly used to extract low-dimensional features for modelling [29]. Specifically, we used transfer learning strategy and a Resnet50 model pretrained on ImageNet to extract feature maps. As a commonly used backbone model, the pre-trained Resnet50 model is able to extract visual representation from image patches [30]. Average pooling was applied to the output feature maps to obtain 1024-dimensional feature vectors [31]. In this way, each 256 × 256 patch was converted into a 1024-dimensional feature vector, making it feasible to simultaneously fit thousands of patches for a single slide into GPU memory.

### Attention-based model building and risk score prediction

The DeepRisk was optimized using the features of all patches of a single case as model inputs and using survival time and survival status as labels. A multiple instance learning (MIL) scheme is used to aggregate the features of all patches of the WSIs for a single case and predict the bag label [32]. The model integrates N patch features of a WSI by using an attention pooling module; by predicting N corresponding attention scores, the attention module enables the model to unambiguously learn which morphological features should be considered prognostic indicators of high risk. Specifically, given a patient with a single slide or multiple slides, we denote instance-level features embedding in the bag with B instances as $\left\{ {f_{i} , i = 1, 2, \ldots ,B} \right\}$. Following the attention module, the attention score of instance ${\text{k}}$ is calculated as follows:

$${a_k} = \frac{{{\text{exp}}\left\{ {w_k^T\left( {{\text{tanh}}\left( {Vf_k^T} \right) \odot sigmoid\left( {Uf_k^T} \right)} \right)} \right\}}}{{\sum\nolimits_{j = 1}^K {{\text{exp}}\left\{ {w_j^T\left( {{\text{tanh}}\left( {Vf_j^T} \right) \odot sigmoid\left( {Uf_j^T} \right)} \right)} \right\}} }}$$

where $U$ and $V$ are learnable parameters, $\odot$is elementwise multiplication, and ${\text{tanh}}\left( \cdot \right)$ and $sigmoid\left( \cdot \right)$ are nonlinearities.

The case-level presentation aggregating all instance embedding features is given by:

$$h_{case} = \mathop \sum \limits_{k = 1}^{B} a_{k}\, f_{k}$$

Then the prediction results are given via the fully connection layer by $p_{case} = W_{c} h_{case}$, where $W_{c}$ is the trainable parameters in fully connection layer. The training process is supervised using the cross entropy-based Cox proportional loss function following for survival analysis (detailed in Additional file 1: Methods S1).

### Integration of multi-scale features and clinical features

Analogous to the diagnostic process used by pathologists to obtain pathological image information in the clinic, DeepRisk contains a hierarchical network to extract WSI features under different magnification scales. Specifically, the WSIs were preprocessed at different magnifications (5 × , 10 × and 20 ×) to extract image patches and corresponding feature embeddings. Then, the attention module corresponding to each magnification aggregated image patches into case-level features. Finally, we concatenated the case-level features at different magnifications and input them to the fully connection (FC) layer for generating DPS. Specifically, the survival time of patients is subdivided into ${\text{n}}$ intervals $\left[ {0,{ }a_{1} } \right),\left[ {a_{1} ,a_{2} } \right), \ldots ,\left[ {a_{n - 1} ,\infty } \right)$, which results in a discrete scale ${\text{t}} = 1,2, \ldots ,{\text{n}}$. And each time point ${\text{t}} \in \left\{ {1, \ldots ,{\text{n}}} \right\}$ is represented by a neuron in the FC layer. Then the survival function can be calculated by

$${\text{S}} = \prod 1 - p_{case}$$

where $p_{case}$ is the discrete hazard of FC layer output. The risk related DPS can be expressed as

$$PRS_{GC} = - \sum S$$

Which is positively correlated with case risk (detailed in Additional file 1: Methods S1). For the fusion of clinical information, we concatenated the gender and normalized age of each case with the corresponding WSI features.

### Visualization of prognosis-related features

To visually interpret the clues that DeepRisk used to predict prognosis from the WSIs, we analysed the clues hierarchically from three perspectives. Firstly, deep features were extracted from the penultimate hidden layer of the model, and t-distributed stochastic neighbor embedding (t-SNE) was used to reduce the dimension of the feature vector to visualize the distribution of each case in the feature space. Second, the attention scores across all patches for each WSI were computed and were converted to percentile scores between 0.0 (low attention) to 1.0 (high attention) (Additional file 1: Fig. S1). Top-scored patches (100 patches for each case) were extracted and their feature embedding was reduced to two dimensions with t-SNE and plotted using hexagonal heat map. Finally, we used QuPath software (version 0.2.3) to segment cells in the patches and trained a support vector machine (SVM) model to identify different cell types. We determined the proportions of different cell types in GC tumour samples from patients with different risk prognoses (for more details, refer to Additional file 1: Methods S1).

### Tumour immune infiltration, differential gene expression and pathway enrichment analysis.

To explore the potential correlation between the DPS and immune cell infiltration, the TCGA-STAD dataset was divided into 2 subgroups: low- and high-DPS groups. As we previously described [33], 22 human immune phenotypes of TCGA-STAD dataset were analyzed based on the CIBERSORT algorithm and the LM22 gene signature. We used the DESeq2 package (version 1.38.0) from Bioconductor in R environment (version 3.6.1, https://www.rproject.org/) to perform differential gene expression analysis, and set P_adj_ < 0.05 and a |log2FC|≥ 1.5 as screening criteria [34]. We next performed the pathway enrichment analysis of those differentially expressed genes by using clusterProfiler V3.19.0 [35] (enricher function, KEGG gene sets, or cancer hallmark gene sets from msigdb). *P* = 0.05 was set as the cut-off value.

### Multiplex Immunohistochemistry (mIHC) and data analysis

To investigate the composition of different immune cells in GC, 12 tissue samples were enrolled and conducted with mIHC analysis, every GC tissue contains normal region, tumor region and invasive margin (IM, 500 µm width on each side of the intra- and peri-tumor interface) [36]. Among these samples, 6 were from high-DPS GCs and another 6 from low-DPS GCs. Consecutive slides from these samples were stained by mIHC and H&E. The mIHC was performed on consecutive slides by using the Opal 7-color kit (Akoya Biosciences) as previously described [37]. Three mIHC panels were employed to characterize different subsets of tumour-infiltrating immune cells (TIICs), including panel 1 (lymphocytes): CD3, CD4, CD8, CD16, CD56 and Foxp3; panel 2 (myeloid cells and B cells): CD11b, CD11c, CD20, CD45RO, CD68, and MPO; panel 3 (immune inhibitory molecules): PD-1, PD-L1, LAG3, TIM3, CTLA4, and IDO. Detailed information of primary antibodies is provided in the Additional file 1: Table S1. The slides were scanned and imaged using a Vectra 3.0 Quantitative Pathology Imaging System (Perkin Elmer, Waltham, MA). Image analysis software, including InForm 2.3 (Perkin Elmer) and HALO (Indica Labs), was used for spectral unmixing, cell segmentation, and the identification and quantification of cellular subsets [38]. The fraction of each lineage of cells was normalized to the number of tumour cells in each analysed field [39].

### Statistical analysis

Percentages or median values are presented as descriptive summary statistics. Pearson’s χ^2^ test or Fisher’s exact test was employed to compare categorical variables. The Wilcoxon rank sum test or Student’s *t* test was used to evaluate continuous variables. OS and disease-free survival (DFS) were estimated by the Kaplan–Meier method and compared using the log-rank test. Cox’s proportional hazards regression model was used to analyze independent prognostic factors. Model performance was assessed with Harrell’s concordance index (C-index). The predictive accuracy of DPS was evaluated by the integrated area under the receiver operating characteristic (ROC) curve (iAUC) with 1000 × bootstrap resampling [40]. Correlation coefficients were computed by Spearman and distance correlation analyses. All correlation heatmaps were generated using the pheatmap function (https://github.com/raivokolde/pheatmap). *p* < 0.05 was considered to indicate statistical significance.

## Results

### Clinicopathological characteristics of patients

Demographics and clinical characteristics of GC patients, including tumour location, tumour differentiation, Lauren type and TNM stage, are summarized in Additional file 1: Table S2. In Zhongshan dataset, death was reported in 215 patients, 37.6% of whom had tumour recurrence, while the recurrence rate was 25.0% among the other 905 patients who survived. The median follow-up duration was 110.8 months (range: 1.5–147.0 months). The 1-, 3- and 5-year overall survival (OS) rates were 94.2%, 77.0% and 70.0%, respectively, and the 1-, 3- and 5-year recurrence rates were 20.0%, 42.5% and 53.9%, respectively.

### Model construction of DeepRisk network

**Fig. 1:**
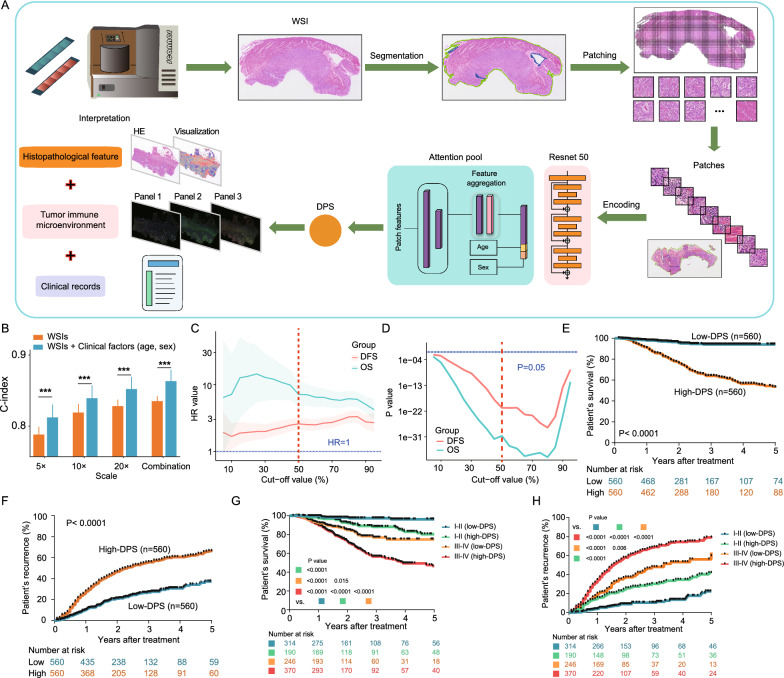
Workflow for DeepRisk model building and evaluation of DPS performance. **A** Underwent segmentation and patching process from WSIs, all the patches were encoded using a deep CNN model into descriptive feature representations. A pre-trained ResNet50 model was used to extract feature maps, to which average pooling was applied to obtain feature vectors. Attention-based MIL was used to aggregate all patch features of a single case and deliver an output label. Then, we built the DeepRisk network without annotation and further validated in 2 external cohorts (TCGA-STAD and SOBC). Histopathological features, immune contexture, transcriptomics and clinical information were used to investigate the correlations between model output (DPS) and underlying GC features. **B** C-indices based on DPS under different magnification scales, with and without incorporation of demographic factors (age and gender). **C**, **D** Performance of DPS in predicting patient survival in the Zhongshan dataset, using a series of DPS cut-off values for patient dichotomising. **E**, **F** Kaplan–Meier curves of OS and DFS for high-DPS (> 50%) and low-DPS (≤ 50%). **G**, **H** Kaplan–Meier curves for survival and recurrence of DPS at different TNM stages. ***p < 0.001. CNN, convolutional neural network; GC, gastric cancer; WSI, whole-slide image; DPS, digital pathology signature; MIL, multi-instance learning; TCGA, The Cancer Genome Atlas

The model was built based on the Zhongshan dataset (n = 1120) and further validated in the TCGA-STAD (n = 268) and SOBC datasets (n = 277). We performed threefold cross-validation, which split cases randomly (1:1:1) in Zhongshan cohort into training set, validation set and testing set, and this was repeated 3 times to obtain all cases results. All WSIs were first preprocessed to extract image patches at different magnifications, and a total of about 18 million 256 × 256 20 × image patches were extracted for further analysis (Fig. 1A). The training process was supervised by patient overall survival time and status. All patch features are integrated by attention scores in a weighted summation to get the case features, and the case risk is predicted by a FC layer. The DPS is the case risk accumulation. Once the training process is finished, in the inference stage the model outputs the DPS based on the input WSIs. C-index via the DPS was used as evaluation metric for the performance of different models. Then, we examined the predictive performance of DeepRisk using the Zhongshan dataset. The DeepRisk model exhibited C-index of 0.781, 0.812 and 0.821 at 5 × , 10 × and 20 × magnification, respectively, based on WSI features alone (Fig. 1B). The combination of the three magnification scales (5 × , 10 × and 20 ×) showed consistently better performance (C-index = 0.828) than each single scale. Furthermore, the incorporation of clinical factors (age and sex) with WSI features significantly improved model performance (C-index) at every magnification scale compared with WSI features only (p < 0.001). The combination of the three scales plus clinical factors achieved a C-index as high as 0.856. These results indicate that DeepRisk effectively captures multiscale patch information, and its performance is further enhanced by adding clinical demographic features.

### Prognostic value of the DPS in Zhongshan dataset

With the DPS as the model output, the patients were dichotomized into high- and low-DPS groups based on various DPS cut-off values. Our model demonstrated significant separation in OS and DFS outcomes across a wide range of DPS cut-off values (ranging from 5 to 95%), with hazard ratios (HRs) > 5 for OS and > 1 for DFS (Fig. 1C, D). Given a relatively even distribution of patients across the continuum of DPS values, 50% was chosen as the cut-off to maximize the number of patients in each risk group. Patients with high-DPS had significantly worse outcomes with a lower survival rate and higher recurrence rate than those with low-DPS (Fig. 1E, F).

**Fig. 2:**
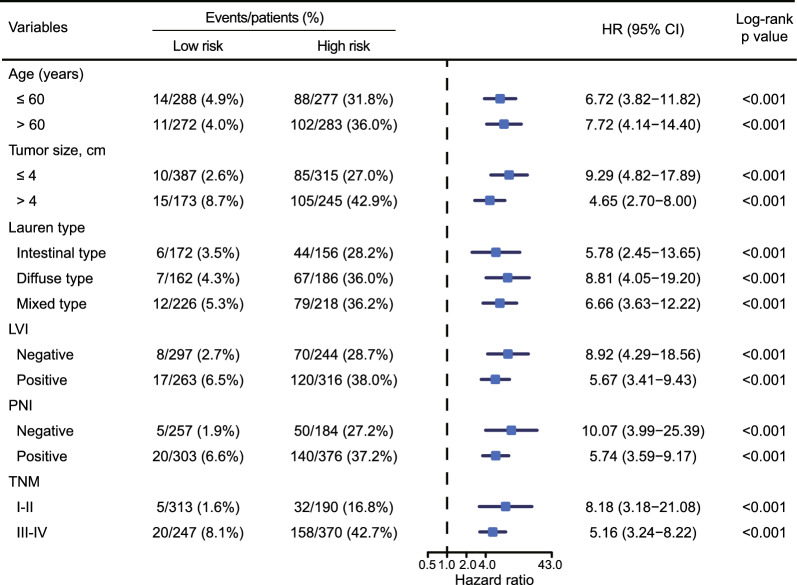
Forest plot of DPS for the Zhongshan dataset in overall survival analysis

Multivariate Cox regression analysis revealed that a high-DPS was significantly associated with unfavourable OS (HR: 5.41, 95% CI 3.55–8.24, p < 0.0001) and DFS (HR: 1.97, 95% CI 1.59–2.44, p < 0.0001), independent of other clinicopathological features (Table 1). Notably, subanalyses stratifying patients into subgroups based on different clinicopathological characteristics consistently demonstrated superior survival outcomes (OS and DFS) for high-DPS patients (Figs. 1G, H, 2 and Additional file 1: Fig. S2), indicating that the DPS strongly correlates with patient prognosis and is nonredundant with other prognosis-related baseline variables.

**Table 1:** Univariate and multivariate cox analysis of disease-free survival and overall survival in the Zhongshan dataset

Variables	Overall survival						Disease-free survival					
	Univariate			Multivariate			Univariate			Multivariate		
	HR	95% CI	*P*	HR	95% CI	*P*	HR	95% CI	*P*	HR	95%	*P*
Age (>60 vs ≤60 years)	1.32	1.01–1.73	0.043	1.18	0.89–1.55	0.245	1.11	0.91–1.34	0.306			
Sex (Female vs male)	0.79	0.59–1.08	0.136				0.80	0.65–1.00	0.045	0.94	0.76–1.17	0.60
Tumor location	1.05	0.88–1.25	0.582				0.90	0.80–1.02	0.099			
Tumor size (>4 vs≤4)	2.74	2.09–3.59	<0.0001	2.11	1.49–2.99	**<0.0001**	2.11	1.74–2.56	<0.0001	1.33	1.09–1.63	**0.005**
Lauren type	1.18	1.00–1.40	0.047	1.05	0.88–1.25	0.61	1.06	0.94–1.20	0.303			
LVI (positive vs negative)	2.31	1.74–3.06	<0.0001	1.39	1.03–1.87	**0.030**	1.92	1.57–2.33	<0.0001	1.26	1.02–1.55	**0.029**
PNI (positive vs negative)	2.45	1.80–3.33	<0.0001	1.44	1.03–2.00	**0.031**	2.13	1.72–2.64	<0.0001	1.28	1.01–1.61	**0.040**
TNM stage (III–IV vs I–II)	5.09	3.57–7.26	<0.0001	2.72	1.83–4.05	**<0.0001**	3.91	3.11–4.91	<0.0001	2.50	1.93–3.23	**<0.0001**
Tumor differentiation (Undifferentiated vs differentiated)	1.03	0.79–1.35	0.83				0.80	0.66–0.97	0.024	0.69	0.57–0.84	**0.0002**
DPS (high vs low)	7.12	4.69–10.81	<0.0001	5.41	3.55–8.24	**<0.0001**	2.58	2.09–3.18	<0.0001	1.97	1.59–2.44	**<0.0001**

LVI, Lymphovascular invasion; PNI, Peripheral nerve invasion; DPS, digital pathology signature; TNM, tumor, node, and metastasis

**Fig. 3:**
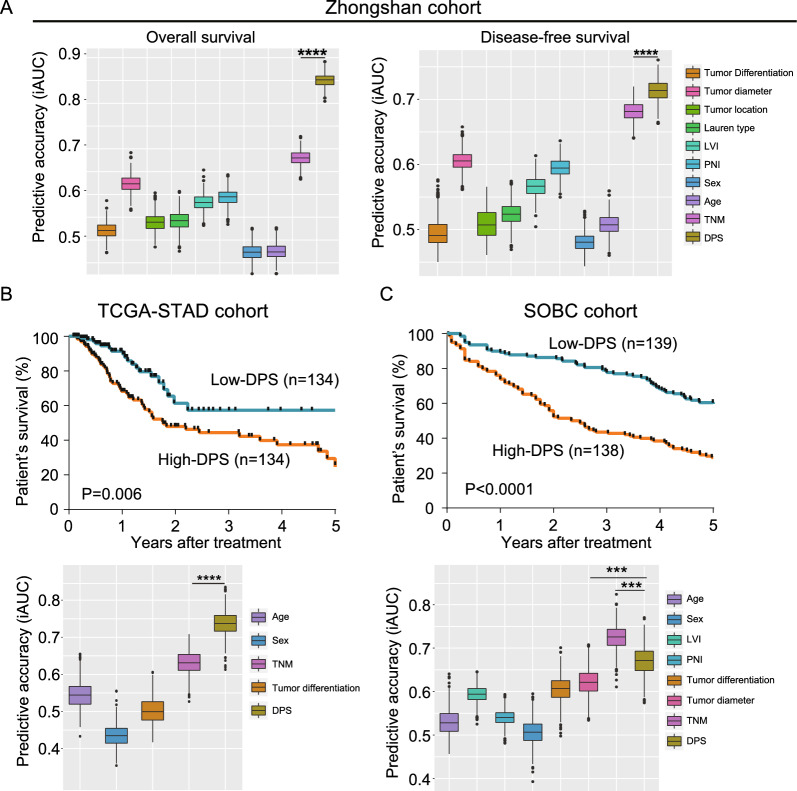
Performance of DPS compared with clinicopathological parameters in three independent datasets. **A** Clinical performance of tumour-related parameters and DPS. The predictive accuracy for survival and recurrence based on the iAUC with 1000 × bootstrap resampling for each parameter is shown in a box plot. **B**, **C** Kaplan–Meier curves and predictive accuracy for overall survival in TCGA-STAD and SOBC cohorts

To evaluate the prognostic accuracy of the DPS, iAUC analysis with 1000 × bootstrap resampling was conducted. The C-index of DPS in predicting OS (0.84, 95% CI: 0.81–0.87) and DFS (0.71, 95% CI: 0.68–0.75) was superior to that of existing clinicopathological parameters (p < 0.0001), including TNM stage, tumour differentiation, tumour location, and Lauren type (Fig. 3A, B). Furthermore, by calculating the continuous net reclassification improvement (NRI) for 5-year postoperative mortality, we combined the TNM stage and the DPS into a single model and compared its predictive ability to TNM stage alone. The combined model containing the DPS showed a continuous NRI of 34.5% (95%CI: 0.209–0.455; p = 0.002), suggesting that the novel DPS may serve as a potential complement to the TNM staging system.

### Validation of the DPS

Next, the DPS was validated in the TCGA-STAD dataset (268 patients with 583 WSIs). After dividing the TCGA-STAD dataset using DPS 50th percentile as a cut-off value, Kaplan–Meier analysis indicated that both groups were well stratified (p = 0.006, Fig. 1F). The C-index of the DPS in predicting OS was 0.74 (95% CI 0.67–0.80). Multivariable Cox analysis remained an independent predictor of OS in the TCGA-STAD dataset (HR: 1.76, 95% CI 1.04–2.98, p = 0.036, Additional file 1: Table S3). Moreover, the predictive power of the DPS was statistically superior to that of age, sex, tumour differentiation and TNM stage (p < 0.0001, Fig. 3C). Although the majority of patients in the TCGA-STAD dataset had a short follow-up duration, TNM stage + DPS had significantly better predictive performance for the 1-year postoperative mortality rate, with an NRI of 0.368 (95% CI 0.074–0.518, p = 0.032). These findings suggest that the DPS could add prognostic value to the TNM staging system. To investigate the prognostic performance of DPS in tissue microarray (TMA) with limited pathological information, the SOBC dataset (277 patients with 552 TMA spots) was also enrolled. Similar trends were observed in the survival stratification (p < 0.0001, Fig. 1G) and prognostic prediction (C-index, 0.67, 95% CI 0.61–0.74; Fig. 3D and Additional file 1: Table S3).

To investigate whether the DPS is superior to existing gene or immune signatures, we compared the DPS with previous prognostic models using the mRNA expression profile of TCGA-STAD [5, 41, 42]. The DPS showed stronger prognostic power for OS (p < 0.001, Additional file 1: Fig. S3) compared to the seven-gene signature, collagen score, and T-effector signature. As expected, our novel AI-based pathological predictor demonstrated better prognostic performance for OS.

### DPS and neoadjuvant chemotherapy

**Fig. 4:**
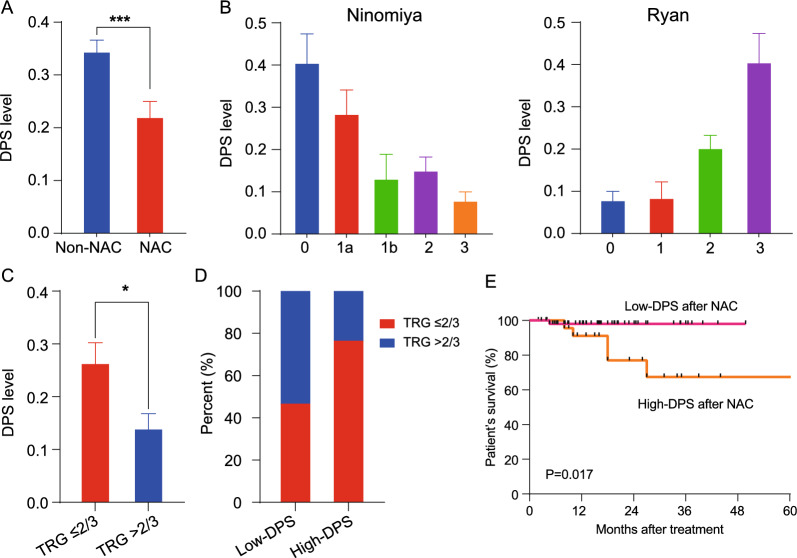
DPS levels was negatively correlated with the treatment response of NAC. **A** Comparison of the DPS levels between Non-NAC and NAC treatment groups; **B**–**D** The relationship between treatment response and DPS levels; **E** Kaplan–Meier survival analysis between patients with low-DPS after NAC and those with high-DPS after NAC treatment. *p < 0.05, ****p < 0.0001. TRG, tissue regression grade; NAC, neoadjuvant chemotherapy

To investigated whether GCs with different DPS level show differential responses to neoadjuvant chemotherapy (NAC), 82 GCs received NAC was enrolled in our study. As shown in Fig. 4A, the DPS level of GCs after receiving NAC was significantly decreased, in contrast to those not receiving NAC treatment. According to the Ninomiya and Ryan classification systems [25, 26], we observed that the DPS level after NAC treatment was negatively associated with tumor regression grade (TRG) (Fig. 4B). Our analysis of GCs with low-DPS after NAC treatment revealed that 53.8% of GCs presented a better treatment response (TRG > 2/3) (Fig. 4C, D). Furthermore, a significant difference was observed between low- and high-DPS patients after receiving NAC treatment (Fig. 4E). Consequently, these findings suggest that DPS after NAC treatment provides potential evaluation efficacy and prognostic value for GC patients, facilitating the subsequent optimization of treatment strategy.

### Feature visualization and interpretation

**Fig. 5:**
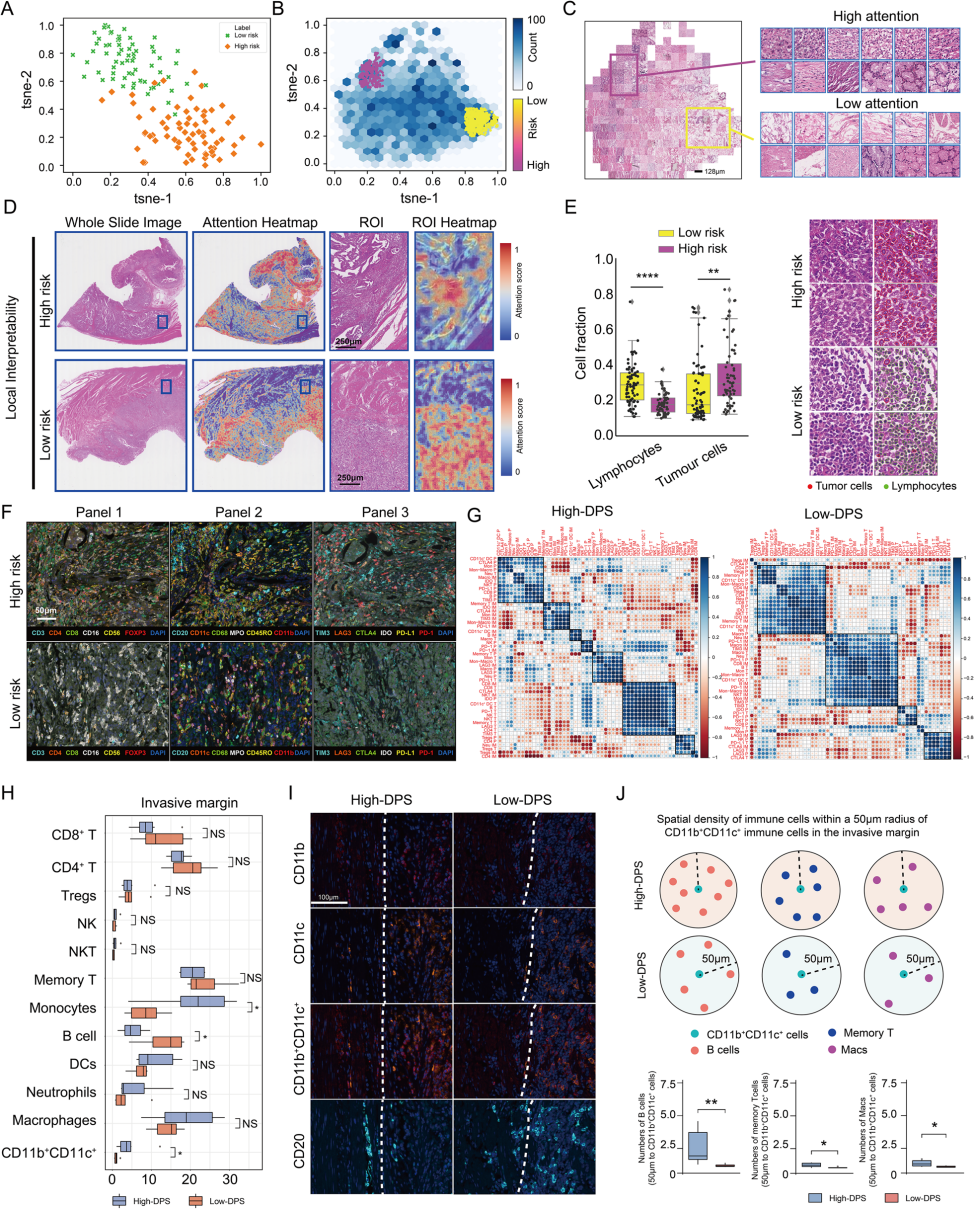
High-DPS associated with suppressive tumor immune microenvironment at the invasive margin. **A** Separability of high- and low-risk area on feature space. **B**–**D** Identification of DPS high-risk and low-risk areas on heatmap. **E** The cell fractions of lymphocytes and tumor cells between low- and high-DPS groups. **F** mIHC panels showing spatial distribution patterns of TIICs on high- and low-DPS specimens. **G** Correlation matrix followed by unsupervised hierarchical clustering of 54 immune features in high- and low-DPS groups. **H** Differences in the density of immune cell types at the invasive margin between high-and low-DPS groups. **I** mIHC expression pattern of CD11b^+^CD11c^+^ immune cells and B cells at the invasive margin. **J** Spatial density of immune cells (memory T cells, B cells and Macs) within a 50 μm radius of CD11b^+^CD11c^+^ immune cells at the invasive margin. Macs, macrophages. TIIC, tumor-infiltrating immune cells. *p < 0.05, **p < 0.01, ****p < 0.0001. Data are represented as mean ± SEM. **p < 0.01, ****p < 0.0001. P, peritumor tissues; IM, invasive margin; T, tumor core

To visualize and interpret the importance of each region in the WSI, whole-slide-level heatmaps were generated by the attention scores as described previously [28]. As shown in Fig. 5A, clear separation of patient-level features between high- and low-DPS patients was observed in the feature space. Pseudocolours on the heatmap show the clustering of high-risk features (purple) and low-risk features (yellow) in the upper left and lower right corners of the heatmap, respectively (Fig. 5B). All the top-ranked patches were retrieved from the high- and low-DPS groups, and reviewed by 2 pathologists independently. Possible annotations of the featured patches were provided. We observed that top patches from the high-DPS cluster mainly contained tumour cells, lymphocytes and dense tumor stroma, while those from the low-DPS cluster mainly contained normal mucosa and loose stroma (Fig. 5C). To identify and determine the proportions of different cell types in GC, the QuPath software was used as previously described [15]. Interestingly, our results revealed higher proportion of tumour cells and lower proportion of lymphocytes in the high-DPS group (Fig. 5D, E), suggesting that the imbalance of tumor immune microenvironment may facilitate tumor progression and metastasis in GC.

### The imbalance of local tumor immune microenvironment associated with high-DPS at the invasive margin

The in situ immune microenvironment plays a critical role in carcinogenesis and distant dissemination [43]. In our study, we performed mIHC with 3 immune panels on the high-DPS (n = 6) and low-DPS (n = 6) specimens (Fig. 5F). The density of 54 immune features in 3 regions of GC was evaluated: normal region, invasive margin and tumor region. Based on correlation analysis, we identified 7 major clusters in the high-DPS group. In the high-DPS group, Fig. 5G illustrated that one cluster in the invasive margin was characterized by myeloid cells and immune inhibitory markers (including memory T, Mon, Mon-Macro, CD11c^+^ DC, TIM3, IDO and CTLA4). Additionally, another one cluster in tumor region the was characterized by lymphocytes and immune inhibitory markers (including CD4, CD8, NK, NKT, B cells, memory T, LAG3, TIM3, CTLA4, IDO and PD-1). These findings suggested an exhausted immune state within the high-DPS group. Then, we compared the immune subsets between high- and low-DPS groups in tumor region and invasive margin, respectively. Interestingly, we observed increased infiltration of CD11b^+^CD11c^+^ immune cells (a phenotype of myeloid derived suppressor cells, MDSCs) [44] and monocytes in tumor region and invasive margin, and a decreased infiltration of B cells at the invasive margin of high-DPS group (Fig. 5H, I and Additional file 1: Fig S4). To explore the relationship between immune cells and tissue architecture, we focused on invasive margin (tumor-immune boundary), which has been previously implicated in playing a prognostic role in tumor progression [36]. Spatial analysis showed a significant increase in B cells, memory T cells and macrophages around CD11b^+^CD11c^+^ immune cells (within 50 μm radius) at the invasive margin of high-DPS group (Fig. 5J). Consistent with previous studies, the potential interaction between CD11b^+^CD11c^+^ cells and other 3 immune cells may promote the imbalance of local tumor immune microenvironment [45].

**Fig. 6:**
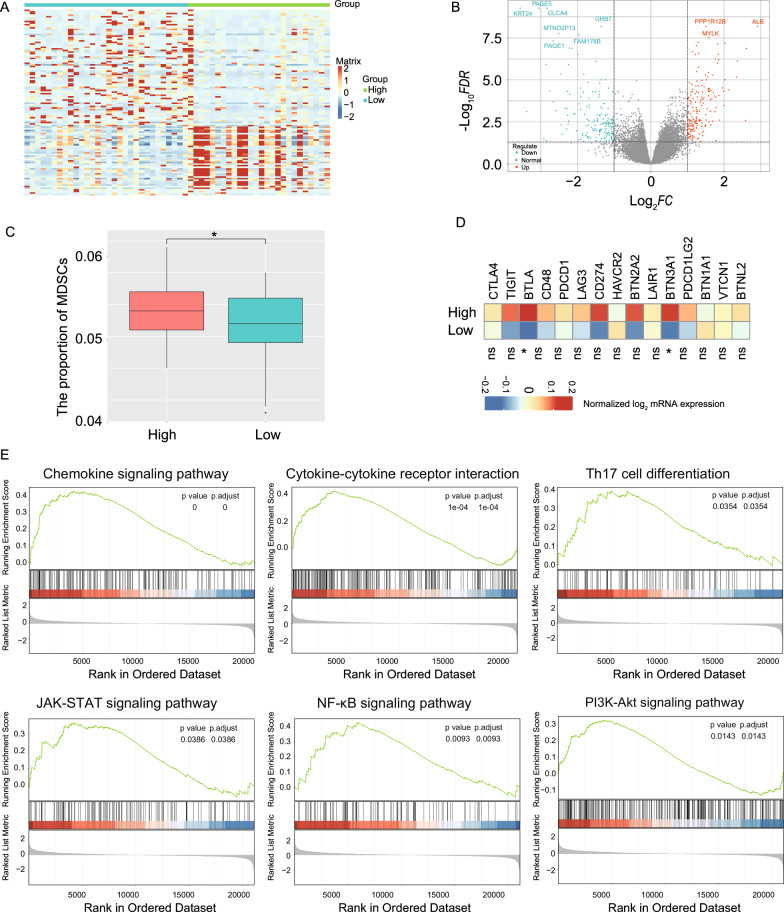
Transcriptomic analysis of tumor suppressive immune microenvironment associated with high-DPS. **A** Heatmap shows the expression levels of differentially expressed genes between high- and low-DPS groups with TCGA-STAD profile. **B** Volcano plot shows the differentially expressed genes in the high-DPS group compared with the low-DPS group. **C** Comparison of the abundance of MDSCs between high- and low-DPS groups; **D** Normalized mRNA expression levels of immune co-inhibitor between low- and high-DPS groups. **E** GSEA results showing the enrichment of six representative pathways in the high-DPS group. MDSCs, myeloid-derived suppressor cells; GSEA, gene set enrichment analysis

To investigate the underlying relevance of pathomics features to molecular mechanism and tumor immune microenvironment, the mRNA expression profile of TCGA-STAD was used in our study. The differentially expressed genes identified between the low- and high-DPS groups were shown in Fig. 6A, B. By using CIBERSORT method with TCGA-STAD data [33], we observed that high-DPS group was characterized by a higher abundance of MDSCs (Fig. 6C). Furthermore, we investigated the expression levels of immune co-inhibitors and found that BTLA and BTN3A1 had higher expression (Fig. 6D), delineating that high-DPS group might escape immune surveillance by MDSCs infiltration and immune co-inhibitors expression. Additionally, we performed Gene Set Enrichment Analysis (GSEA) to identify gene expression signatures that might be correlated with DPS status. Figure 6E revealed the activation of the chemokine signaling pathway, cytokine-cytokine receptor interaction, Th17 cell differentiation, JAK-STAT signaling pathway, NF-κB signaling pathway, and PI3K-AKT signaling pathway, implicated in immune regulation [46], cancer progression [47] and metastasis [48].

## Discussion

Though cancer prognostication and precise treatment are increasingly driven by biomarkers in histology, clinical data and genomics, pathomics remains a cornerstone of oncology. In this study, we developed a weakly supervised DeepRisk model for GC based on WSIs. Model development included adopting the process of expanding magnification scales, which mimics the way in which pathologists examine histopathological images, and utilizing an attention-based mechanism to focus on image regions important for patient prognosis, without any manual annotation. Our findings demonstrate that this model is a robust tool for GC prognostic evaluation and is applicable to various types of datasets (WSIs and TMAs) obtained from different cohorts and processed following different protocols. As the model output, a novel artificial intelligence (AI)–based pathomic score, the DPS, was developed. Our results suggest that the DPS is independent of and stronger than that of a variety of clinicopathological features (including TNM stage system), and may be a potential supplement to the TNM staging system for GC prognosis prediction and patient stratification.

Because of the ‘black box’ nature of neural networks [49], the lack of interpretability has become a major limitation that hinders the clinical application of deep learning models. In this study, we interpreted the model hierarchically from case-, patch- and cell-level, and visualized the feature embeddings of cases with different prognostic risks via t-SNE dimensionality reduction. The good separability of high- and low-risk labels in the t-SNE space illustrated the informativeness of feature embeddings converted from WSI patches using our model. Similarly, the top-scoring patches of cases with different prognostic risks were extracted, and the separability of these patches in the t-SNE space indicated that different patterns of patches dominated the model prediction. These visualizations demonstrate that the model is able to automatically locate regions crucial for prognosis prediction from WSIs and extract informative features from these regions. Our model eliminates the need for manual annotation, which reduces human intervention and therefore generalizes better. The multi-scale approach used in our model is more in line with the pathologist's diagnostic process. According to the comparison results of our mode with several advanced models in Table S4, our model achieved the better performance [9, 11, 12, 50]. Therefore, our method could automate many time-consuming and repetitive tasks, easing the growing workload of clinicians and pathologists.

Recently, Huang et al. developed two deep learning algorithms that were able to identify tumour regions, extract the most suspicious tiles, and generate risk scores for GC diagnosis and prognosis based on digital histopathology derived from TMAs [51]. However, our study has several advantages, including multi-scale sampling, spatial immune contexture analysis, and multi-omics analysis. In our study, we developed a DeepRisk model based on WSIs from GC tissue specimens, which provide more abundant information than TMAs (1–1.5 mm in diameter for each spot). Furthermore, we enrolled a larger sample size in our study, and our DPS achieved excellent performance in the Zhongshan and TCGA-STAD datasets, surpassing any other clinicopathological risk parameters. Our findings are consistent with previous studies showing depleted B cells and increased CD11b^+^CD11c^+^ immune cells at the invasive margin across high-DPS patients, suggesting that the invasive margin is a unique site of immune inhibition with altered expression profiles of immune cells [44, 52]. Previous studies reported that CD11b^+^CD11c^+^ myeloid cells could impair the T cell-stimulating ability by arginase-1, lysosomal protease and COX-2 [53], promote the distant metastasis via CCL2/CCR2 in colorectal cancer [54] and suppress the tumor immune microenvironment and tumor growth through CSF1R/PD-L1 upregulation [55]. Wang et al. also reported that MDSCs suppress B cell proliferation in vitro in an arginase-dependent manner and promote tumor escape from immune surveillance [45]. Interestingly, we also observed the activation of chemokine signaling pathway, cytokine-cytokine receptor interaction, JAK-STAT signaling pathway in the high-DPS group. The above results suggested that the imbalance of the tumor immune microenvironment may influence the immune status under different DPS states.

Neoadjuvant chemotherapy is a common treatment for patients with GC [56]. However, a considerable proportion of GCs do not respond well to NAC [57]. In clinical practice, it is challenging for clinicians to extract comprehensive prognosticators or treatment-related features directly from pathological images. Herein, we proposed a clinical applicable method to simplify this process and aid clinicians in treatment decision-making. Our results indicated a significant decrease of DPS levels in patients responding to NAC, and GCs with low-DPS after NAC could obtain better survival benefit, implying that DPS might be a dynamic parameter for evaluating the efficacy of NAC. Further, our proposed DPS could reflect the intrinsic characteristics of the tumor biology, immune contexture and aggressiveness, which may be associated with response and outcomes to neoadjuvant chemotherapy. The underling mechanism between pathological features and chemotherapy have not been thoroughly elucidated, and further investigation may provide insight and strategies for treatment [11, 19].

There are several limitations to our study. Firstly, due to its retrospective nature, further prospective validation is necessary before our model and the DPS can be routinely applied in clinical settings. Secondly, the study cohorts (Zhongshan and SOBC cohorts) mainly consisted of Chinese population, while the TCGA-STAD cohort included populations from Caucasian (167, 62.3%), African (11, 4.1%) and Asian (42, 15.7%). Thus, further validation with various population is needed. Thirdly, our attention-based case-level feature aggregation method discarded the spatial information of the patches, which may reduce model performance, although our attention-based feature aggregation method still achieved better C-indexes than two previously reported WSI-based networks at multiple magnification scales (Additional file 1: Table S5). Finally, the lack of mechanistic insights is a major limitation. The biological mechanisms and immune contexture underlying DPS remain to be further investigated in order to understand the roles of CD11b^+^CD11c^+^ cells in tumor progression and the interactions between CD11b^+^CD11c^+^ cells and other immune cells (such as B cells and macrophages) in high-DPS GCs.

## Conclusion

In conclusion, our study developed a DeepRisk model and DPS score based on WSIs for GC prognostic evaluation and adjuvant treatment. Our findings suggest that the DPS is a novel AI-based pathomics signature that may supplement the TNM stage system and lead to potential individualized treatment in clinical practice.

### Supplementary Information

**Additional file 1: Methods S1.** **Table S1.** Antibody sources and staining conditions. **Table S2.** Demographic, clinical, and tumor characteristics of patients with gastric cancer in the Zhongshan, TCGA-STAD and SOBC dataset. **Table S3.** Univariate and multivariate Cox analysis of overall survival in the TCGA-STAD and SOBC datasets. **Table S4.** Comparison of C-index of different models on different datasets. **Table S5.** C-index performance comparison of different feature aggregation methods on ZhongShan cohort. **Figure S1.** Heatmaps generated based on WSIs from the Zhongshan dataset. Distribution features of intratumoral fibroblast and extracellular matrix were investigated through α-SMA staining and Masson staining, respectively. **Figure S2.** Forest plot of DPS in the Zhongshan dataset in disease-free survival analysis. **Figure S3.** ROC curves of overall survival (OS) for the DPS and other 3 prognostic models with TCGA-STAD mRNA profile. **Figure S4.** Differences in the density of immune cell types at the tumor region between high-and low-DPS groups.

## Data Availability

The data from Zhongshan Hospital supporting the findings are available upon reasonable request from the corresponding author (XFW) due to patient privacy concerns. The external validation of TCGA data set is publicly available at the TCGA portal (https://portal.gdc.cancer.gov). We have uploaded our source code to the Github repository with site (https://github.com/yyyzzzhao/DeepRisk). The TCGA-STAD dataset can be downloaded from (https://portal.gdc.cancer.gov/projects/TCGA-STAD).

## Abbreviations

GC: Gastric cancer
DPS: Digital pathology signature
WSI: Whole-slide image
H&E: Haematoxylin and eosin
MSI: Microsatellite instability
TCGA: The Cancer Genome Atlas
SOBC: Shanghai Outdo Biotech Company
OS: Overall survival
DFS: Disease-free survival
STAD: Stomach adenocarcinoma
TMA: Tissue microarray
CNN: Convolutional neural network
MIL: Multiple instance learning
t-SNE: T-distributed stochastic neighbour embedding
SVM: Support vector machine
TIIC: Tumour-infiltrating immune cells
