# Supplementary material for: DeepRisk network: an AI-based tool for digital pathology signature and treatment responsiveness of gastric cancer using whole-slide images
Source: J Transl Med. 2024 Feb 19;22:182. doi: 10.1186/s12967-023-04838-5 (PMC10877826; doi:10.1186/s12967-023-04838-5)
Supplement: Supplementary file 1 — Additional file 1: Methods S1. Table S1. Antibody sources and staining conditions. Table S2. Demographic, clinical, and tumor characteristics of patients with gastric cancer in the Zhongshan, TCGA-STAD and SOBC dataset. Table S3. Univariate and multivariate Cox analysis of overall survival in the TCGA-STAD and SOBC datasets. Table S4. Comparison of C-index of different models on different datasets. Table S5. C-index performance comparison of different feature aggregation methods on ZhongShan cohort. Figure S1. Heatmaps generated based on WSIs from the Zhongshan dataset. Distribution features of intratumoral fibroblast and extracellular matrix were investigated through α-SMA staining and Masson staining, respectively. Figure S2. Forest plot of DPS in the Zhongshan dataset in disease-free survival analysis. Figure S3. ROC curves of overall survival (OS) for the DPS and other 3 prognostic models with TCGA-STAD mRNA profile. Figure S4. Differences in the density of immune cell types at the tumor region between high-and low-DPS groups. [file 12967_2023_4838_MOESM1_ESM.docx]

**Methods S1**

**1 DPS calculation and training process**

The Attention module encodes the input patch features to a case-level feature vector with a dimension of 1024, which is then input to the fully connected layer for final classification. Specifically, to calculate the risk score for patients, we divided patient survival time into intervals with fixed boundaries. The discrete results $t=1, 2,\ldots,q$ output by the fully connected layer (followed by a sigmoid activation function) represent the hazard $h$ of cases in each discrete time interval. And the survival function $S$ is defined by

$$S=\prod_{s=1}^{t} (1-h_{t})$$

Where $S$ is a vector of length $q$, which represents the probability that the case survival time exceeds the discrete time point $t$. Accordingly, the DPS is defined by

$${PRS}_{GC}=-\sum S$$

For succinctness, we normalize the range of ${PRS}_{GC}$ to 0~1, which is a value that is positively correlated with case risk.

To optimize the model parameters, we used the cross-entropy loss function, defined by

$$L=-\sum_{(x^{i},t^{i})\in\mathcal{D}_{train}} \{c^{i}\log\left( S\left( t^{i} | x^{i} \right) \right)+(1-c^{i})log(1-S\left( t^{i} | x^{i} \right))\}$$

Where $x^{i}$ is the case-level features and $t^{i}$ is the case observed event times in the training data $\mathcal{D}_{train}$. The binary variable $c^{i}\in\{0,1\}$ indicates whether the observed event time is right censored ($c^{i}=1$) or not ($c^{i}=0$).

We used Adam optimizer with weight decay of 1E-5. The batch-size was 1 and the learning rate was 1E-4. The maximum epoch was 200 and an early stop strategy was used with patience of 20.

**2 Cell counting method**

In order to count the number of each type of cells in image patches with top-ranked attention score, we first need to segment the cells from image patches, and then classify the extracted cells. For the cell segmentation task, instead of using a CNN model that requires manual annotation, we use QuPath (v 0.2.3) software to segment the cells and extract features of cells. We segmented a total of 43702 cells from image patches and for each cell entry, we extracted 53 features including location, shape, color, etc. To train the SVM model for cell classification, we annotated a subset of cells. We skillfully find some pure image patches containing only a single type of cells and label the category, then all cells in the image patch are the labels of the image patch. We trained an SVM model using the labeled 2016 cell entries, and used this model to classify and count all cells.

**Table S1. Antibody sources and staining conditions.**

| Panel | Markers | Antibody source | Species | Clone | Dye | Dilution |
| --- | --- | --- | --- | --- | --- | --- |
| Panel 1 | CD3 | Abcam | Rabbit monoclonal | SP7 | 480 | 1:1000 |
|  | CD4 | Cell Signaling Technology | Rabbit monoclonal | EP204 | 620 | 1:200 |
|  | CD8 | Cell Signaling Technology | Rabbit monoclonal | D8A8Y | 520 | 1:200 |
|  | FOXP3 | Cell Signaling Technology | Rabbit monoclonal | D6O8R | 690 | 1:100 |
|  | CD56 | Cell Signaling Technology | Mouse monoclonal | 123C3 | 570 | 1:400 |
|  | CD16 | Abcam | Rabbit monoclonal | SP175 | 780 | 1:2000 |
| Panel 2 | CD20 | Cell Signaling Technology | Rabbit monoclonal | E7B7T | 480 | 1:1000 |
|  | CD11c | Abcam | Rabbit monoclonal | EP1347Y | 620 | 1:2500 |
|  | CD68 | Cell Signaling Technology | Rabbit monoclonal | D4B9C | 520 | 1:3000 |
|  | CD11b | Abcam | Rabbit monoclonal | EP1345Y | 690 | 1:5000 |
|  | CD45RO | Cell Signaling Technology | Mouse monoclonal | UCHL1 | 570 | 1:400 |
|  | MPO | Cell Signaling Technology | Rabbit monoclonal | E1E7I | 780 | 1:200 |
| Panel 3 | TIM3 | Cell Signaling Technology | Rabbit monoclonal | D5D5R™ | 480 | 1:400 |
|  | LAG3 | Cell Signaling Technology | Rabbit monoclonal | D2G4O™ | 620 | 1:400 |
|  | CTLA4 | Abcam | Rabbit monoclonal | CAL49 | 520 | 1:1500 |
|  | PD-1 | Cell Signaling Technology | Rabbit monoclonal | D4W2J | 690 | 1:400 |
|  | PD-L1 | Cell Signaling Technology | Rabbit monoclonal | E1L3N^®^ | 570 | 1:200 |
|  | IDO | Abcam | Rabbit monoclonal | SP260 | 780 | 1:500 |

**Table S2. Demographic, clinical, and tumor characteristics of patients with gastric cancer in the Zhongshan, TCGA-STAD and SOBC dataset.**

| Variables | Zhongshan dataset (n=1120) | | TCGA-STAD dataset (n=268) | | SOBC dataset (n=277) | |
| --- | --- | --- | --- | --- | --- | --- |
|  | N | % | N | % | N | % |
| Sex No. (%) |  |  |  |  |  |  |
| Male | 783 | 69.8% | 182 | 67.9% | 193 | 69.7% |
| Female | 337 | 30.1% | 86 | 32.1% | 84 | 31.3% |
| Age, year |  |  |  |  |  |  |
| ≤60 | 565 | 50.4% | 85 | 31.7% | 103 | 37.2% |
| ＞60 | 555 | 49.5% | 183 | 68.3% | 174 | 62.8% |
| Tumor size, cm |  |  |  |  |  |  |
| ≤4 | 702 | 62.6% | / | / | 122 | 44.0% |
| ＞4 | 418 | 37.3% | / | / | 155 | 56.0% |
| Tumor location |  |  |  |  |  |  |
| Cardia | 206 | 18.4% | / | / | / | / |
| Body | 306 | 27.3% | / | / | / | / |
| Antrum | 583 | 52.0% | / | / | / | / |
| Whole | 25 | 2.2% | / | / | / | / |
| Differentiation status |  |  |  |  |  |  |
| Well + moderate | 510 | 45.5% | 110 | 41.0% | 157 | 56.7% |
| Poor and undifferentiated | 610 | 54.5% | 158 | 59.0% | 120 | 43.3% |
| LVI |  |  |  |  |  |  |
| Negative | 541 | 48.3% | / | / | 233 | 84.1% |
| Positive | 579 | 51.7% | / | / | 44 | 15.9% |
| PNI |  |  |  |  |  |  |
| Negative | 441 | 39.4% | / | / | 246 | 88.8% |
| Positive | 679 | 60.6% | / | / | 31 | 11.2% |
| Lauren type |  |  |  |  |  |  |
| Intestinal type | 328 | 29.3% | / | / | / | / |
| Diffuse type | 348 | 31.1% | / | / | / | / |
| Mixed type | 444 | 39.6% | / | / | / | / |
| Depth of invasion |  |  |  |  |  |  |
| T1 + T2 | 341 | 30.4% | 85 | 31.7% | 51 | 18.4% |
| T3 + T4 | 779 | 69.6% | 183 | 68.3% | 226 | 81.6% |
| Lymph node metastasis |  |  |  |  |  |  |
| N1 | 346 | 30.9% | 71 | 26.5% | 84 | 30.3% |
| N1 + N2 + N3 | 774 | 69.1% | 197 | 73.5% | 193 | 69.7% |
| TNM Stage |  |  |  |  |  |  |
| I + II | 503 | 44.9% | 122 | 45.5% | 108 | 39.0% |
| III + IV | 617 | 55.1% | 146 | 54.5% | 169 | 61.0% |

LVI, Lymphovascular invasion; PNI, Peripheral nerve invasion;

**Table S3. Univariate and multivariate Cox analysis of overall survival in the TCGA-STAD and SOBC datasets.**

|  | Overall survival | | | | | |
| --- | --- | --- | --- | --- | --- | --- |
| Variables | Univariable | | | Multivariable | | |
|  | HR | 95% CI | *P* | HR | 95% CI | *P* |
| **TCGA-STAD dataset (n=268)** |  |  |  |  |  |  |
| Sex (Female vs. male) | 0.64 | 0.99-1.04 | 0.109 |  |  |  |
| Age (>60 vs. ≤60 years) | 1.45 | 0.87-2.44 | 0.156 |  |  |  |
| Histology (Undifferentiated vs. differentiated) | 1.32 | 0.83-2.10 | 0.24 |  |  |  |
| TNM stage (III-IV vs. I-II) | 2.28 | 1.36-3.80 | 0.002 | 2.03 | 1.20-3.42 | **0.008** |
| (high vs. low) | 2.04 | 1.22-3.42 | 0.007 | 1.76 | 1.04-2.98 | **0.036** |
| **SOBC dataset (n=277)** |  |  |  |  |  |  |
| Sex (Female vs. male) | 1.07 | 0.77-1.49 | 0.67 |  |  |  |
| Age (>60 vs. ≤60 years) | 0.99 | 0.73-1.36 | 0.97 |  |  |  |
| Histology (Undifferentiated vs. differentiated) | 1.96 | 1.44-2.66 | <0.0001 | 1.56 | 1.14-2.15 | **0.006** |
| Tumor size (>4 vs.≤4) | 2.14 | 1.55-2.95 | <0.0001 | 1.79 | 1.28-2.50 | **<0.0001** |
| LVI (positive vs. negative) | 3.09 | 2.14-4.45 | <0.0001 | 1.57 | 1.05-2.35 | **0.029** |
| PNI (positive vs. negative) | 1.87 | 1.21-2.88 | 0.005 | 1.19 | 0.76-1.87 | 0.45 |
| TNM stage (III-IV vs. I-II) | 4.13 | 2.83-6.04 | <0.0001 | 3.31 | 2.24-4.90 | **<0.0001** |
| (high vs. low) | 2.29 | 1.67-3.13 | <0.0001 | 2.10 | 1.50-2.94 | **<0.0001** |

**Table S4. Comparison of C-index of different models on different datasets.**

| Models | ZhongShan cohort | TCGA-STAD | SOBC |
| --- | --- | --- | --- |
| Campanella et al[1] | 0.812 | 0.725 | 0.651 |
| Chen et al[2] | 0.793 | 0.709 | 0.613 |
| Saillard et al[3] | 0.798 | 0.720 | 0.633 |
| Shi et al[4] | 0.832 | 0.738 | 0.669 |
| Ours | 0.856 | 0.741 | 0.672 |

**Table S5. C-index performance comparison of different feature aggregation methods on ZhongShan cohort.**

| Magnification | Attention (ours) | GCN [5] | RNN[1] |
| --- | --- | --- | --- |
| 20× | **0.821** | 0.798 | 0.782 |
| 10× | **0.812** | 0.772 | 0.753 |
| 5× | **0.781** | 0.755 | 0.738 |


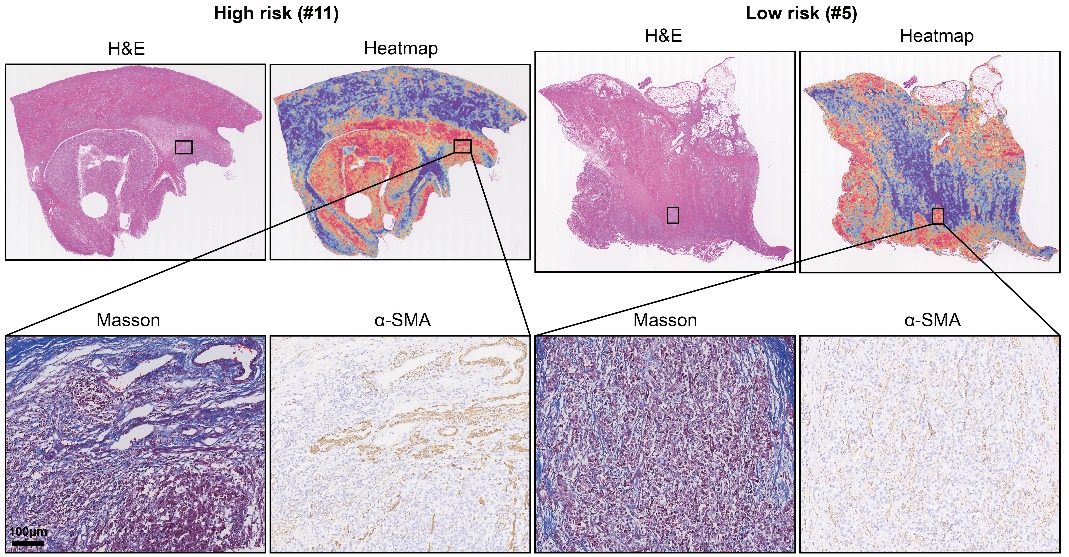


**Fig. S1** Heatmaps generated based on WSIs from the Zhongshan dataset. Distribution features of intratumoral fibroblast and extracellular matrix were investigated through α-SMA staining and Masson staining, respectively.


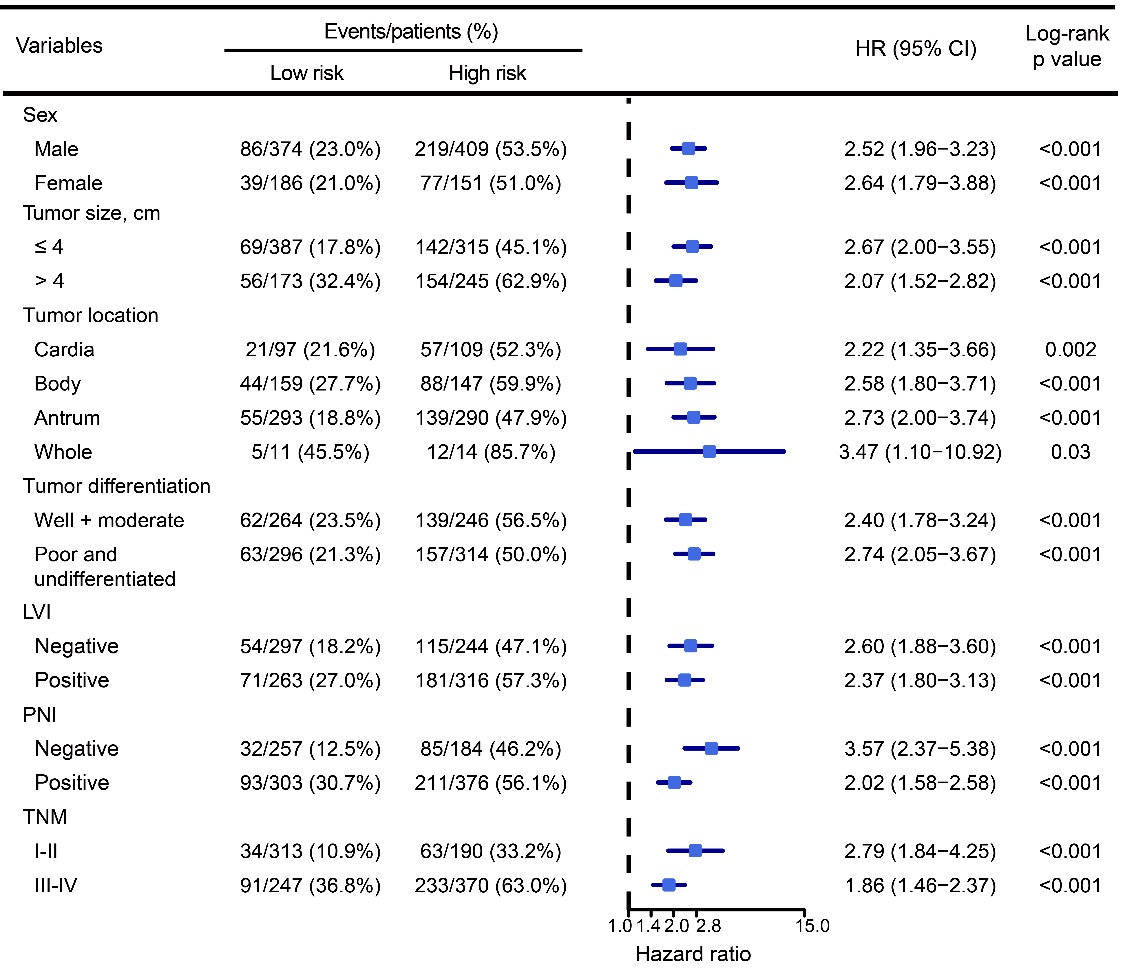


**Fig. S2** Forest plot of DPS in the Zhongshan dataset in disease-free survival analysis.


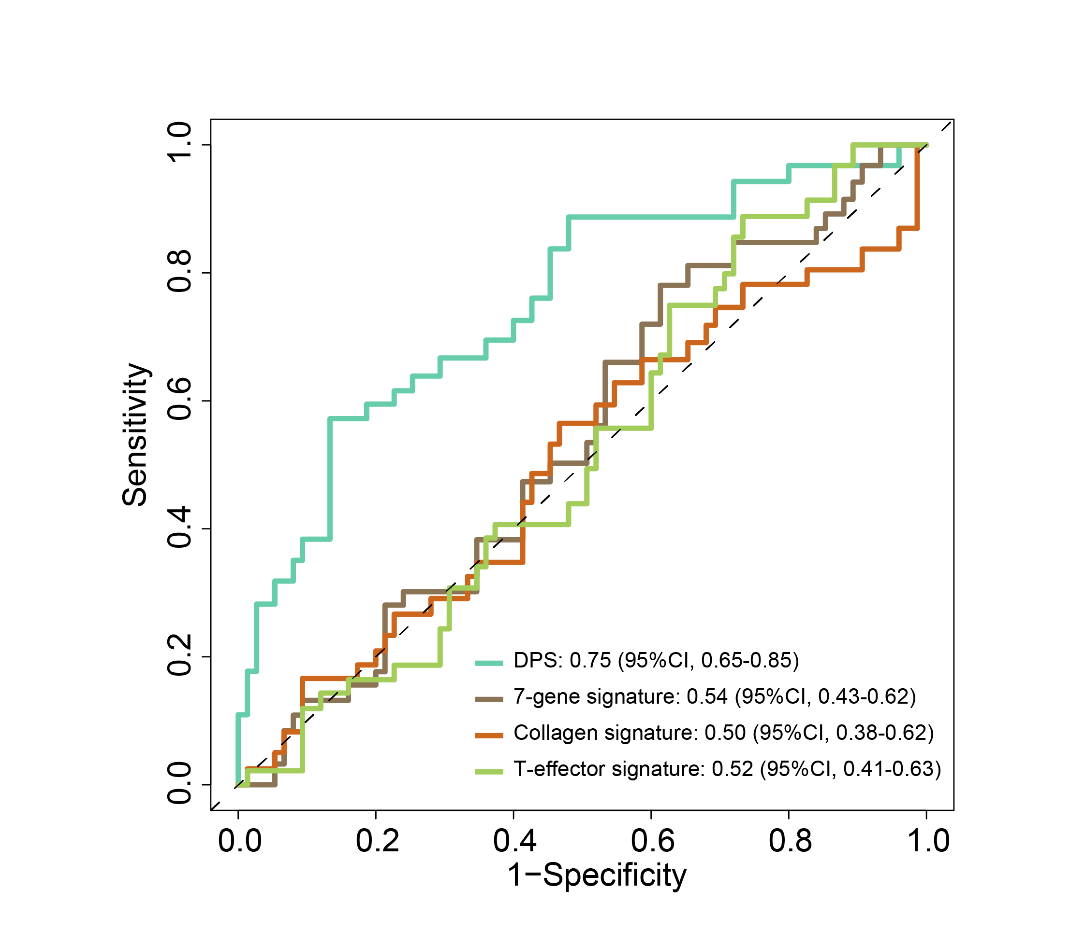


**Fig. S3** ROC curves of overall survival (OS) for the DPS and other 3 prognostic models with TCGA-STAD mRNA profile.


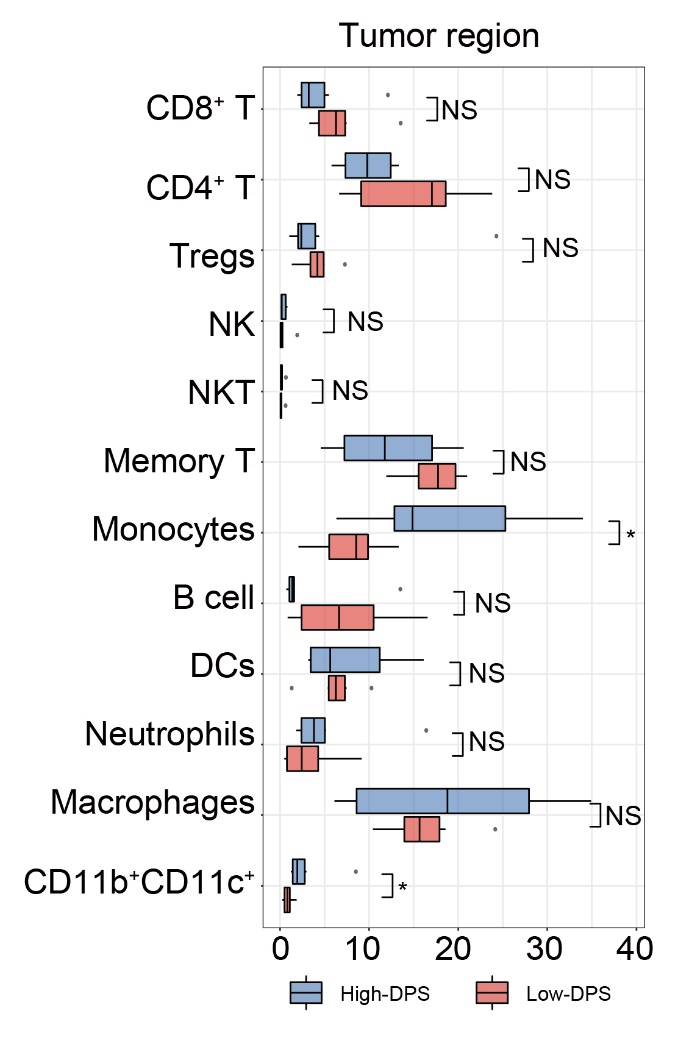


**Fig. S4** Differences in the density of immune cell types at the tumor region between high-and low-DPS groups.

**References**

1. Campanella G, Hanna MG, Geneslaw L, Miraflor A, Werneck Krauss Silva V, Busam KJ, Brogi E, Reuter VE, Klimstra DS, Fuchs TJ: **Clinical-grade computational pathology using weakly supervised deep learning on whole slide images**. *Nature medicine* 2019, **25**(8):1301-1309.

2. Chen D, Fu M, Chi L, Lin L, Cheng J, Xue W, Long C, Jiang W, Dong X, Sui J *et al*: **Prognostic and predictive value of a pathomics signature in gastric cancer**. *Nat Commun* 2022, **13**(1):6903.

3. Saillard C, Schmauch B, Laifa O, Moarii M, Toldo S, Zaslavskiy M, Pronier E, Laurent A, Amaddeo G, Regnault H *et al*: **Predicting Survival After Hepatocellular Carcinoma Resection Using Deep Learning on Histological Slides**. *Hepatology (Baltimore, Md)* 2020, **72**(6):2000-2013.

4. Shi J-Y, Wang X, Ding G-Y, Dong Z, Han J, Guan Z, Ma L-J, Zheng Y, Zhang L, Yu G-Z *et al*: **Exploring prognostic indicators in the pathological images of hepatocellular carcinoma based on deep learning**. *Gut* 2020.

5. Chen RJ, Lu MY, Shaban M, Chen CK, Chen TY, Williamson DFK, Mahmood F: **Whole Slide Images are 2D Point Clouds: Context-Aware Survival Prediction Using Patch-Based Graph Convolutional Networks**. *Medical Image Computing and Computer Assisted Intervention - Miccai 2021, Pt Viii* 2021, **12908**:339-349.
